# Case report: Bilateral pleural effusion secondary to late migration of a tunneled central venous catheter in a patient affected by high risk neuroblastoma

**DOI:** 10.3389/fped.2022.947351

**Published:** 2022-08-04

**Authors:** Tommaso Domenico D'Angelo, Giorgio Persano, Alessandro Crocoli, Cristina Martucci, George Koshy Parapatt, Gian Luigi Natali, Alessandro Inserra

**Affiliations:** ^1^Surgical Oncology – General and Thoracic Surgery Unit, Department of Surgery, Bambino Gesù Children's Hospital Istituto di Ricerca e Cura a Carattere Scientifico (IRCCS), Rome, Italy; ^2^Radiology Unit, Department of Diagnostic Imaging, Bambino Gesù Children's Hospital Istituto di Ricerca e Cura a Carattere Scientifico (IRCCS), Rome, Italy

**Keywords:** central venous catheter complication, central venous catheter migration, bilateral pleural effusion, case report, late central venous catheter mechanical complication

## Abstract

The insertion of long-term central venous catheters is a standard of care for children affected by malignancies, although it can be associated with life-threatening complications. The present paper reports an unusual mechanical complication related to the use of a long term tunneled central venous catheter in a pediatric oncologic patient. An 18 months old child, diagnosed with stage M high-risk retroperitoneal neuroblastoma, underwent ultrasound-guided placement of a 6 Fr bilumen long-term tunneled central venous catheter in the right internal jugular vein prior to the beginning of induction chemotherapy. The correct position of the distal tip of the catheter was confirmed by fluoroscopy. After 4 months of regular use of the device, the patient experienced neck swelling during high-dose chemotherapy infusion. A chest x-ray showed a dislocated catheter and bilateral pleural effusion. CT scan demonstrated the tip of the catheter rupturing the medial wall of the right jugular vein and entering the mediastinum; furthermore, pneumomediastinum, subcutaneous neck emphysema and bilateral pleural effusion were noticed and a thrombus was evident in the right jugular vein at the insertion in the brachiocephalic vein. The patient was then transferred to the Intensive Care Unit and bilateral thoracostomy tubes were placed urgently (500 mL of clear fluid were evacuated from pleural spaces). The dislocated catheter was removed electively on the following day under fluoroscopy. Despite ultrasound-guided placement and long-term uneventful use of the catheter, life-threatening central venous catheter-related mechanical complications can occur; the current case report emphasizes the importance of careful monitoring of patients with central venous catheters in order to quickly diagnose and treat potentially lethal complications.

## Introduction

For children with cancer, the insertion of long-term central venous catheters is standard of care (1, 2). Recent evidence suggests that the use of ultrasound guidance for venous catheter placements minimizes the risk of mechanical complications, such as dislodgment or unintentional pleural puncture (3, 4); despite these precautions, such complications may still occur.

In the present case report, the authors describe a case of central venous catheter dislodgment with associated intrapleural extravasation, occurred 4 months of regular use of the device.

## Case description

**Figure 1:**
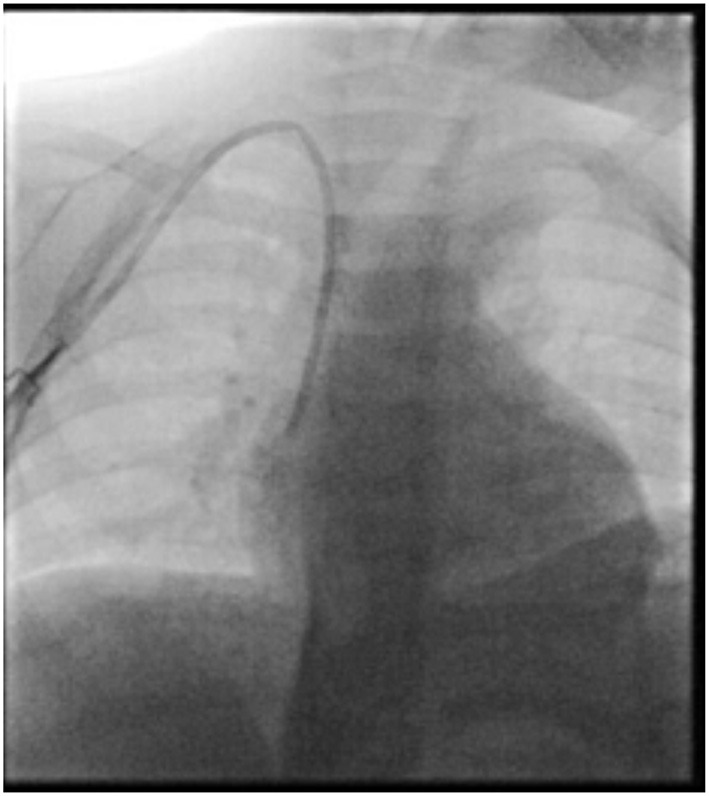
Intra-procedural fluoroscopy.

An 18 months old child was diagnosed with stage M high-risk retroperitoneal neuroblastoma. As per institutional protocol and according to current national and international guidelines (2, 5, 6), after RaCeVA evaluation (7) a 6 Fr bilumen cuffed 3rd generation polyurethane tunneled central venous catheter was positioned in the caudal third of right internal jugular vein by an experienced interventional radiologist, prior to the beginning of induction chemotherapy. The procedure was carried out under ultra-sound guidance, with fluoroscopy confirming the correct position of the catheter's distal tip. The catheter was secured in place with a subcutaneously anchored securement device (Figure 1).

The device worked properly for the whole period (4 months) of induction therapy; during this time, the patient had no local trauma, and repeated radiological examinations, performed to assess chemotherapy response, verified the correct position of the catheter tip.

**Figure 2:**
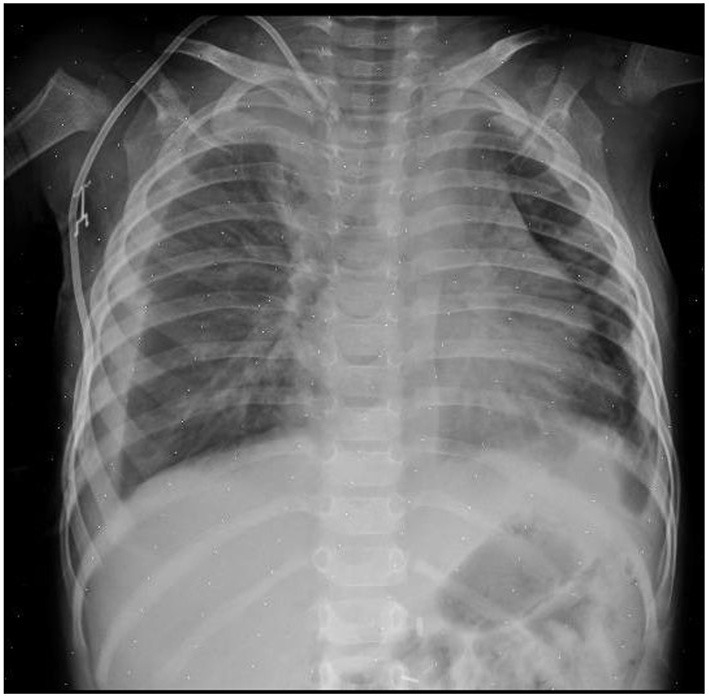
Chest x-ray showing dislocated tip of the catheter and bilateral pleural effusion.

**Figure 3:**
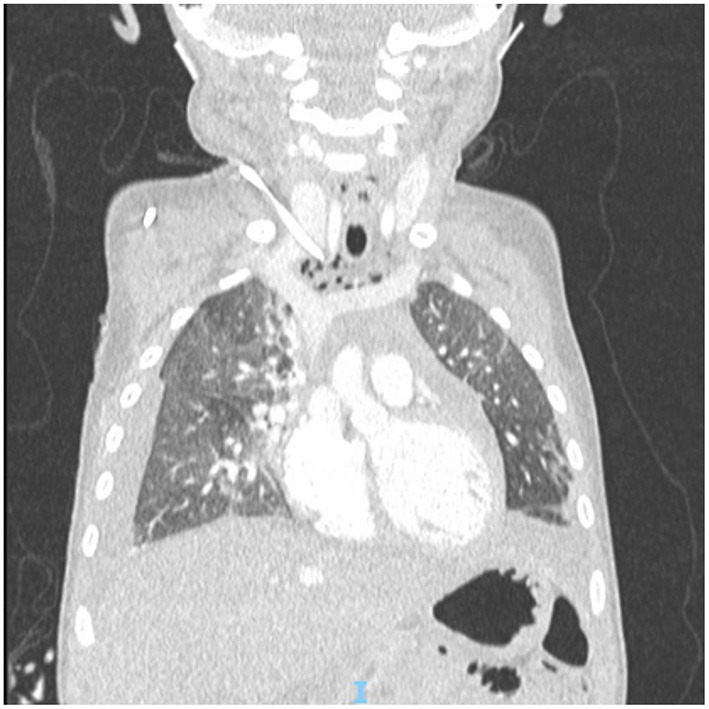
CT scan showing dislocated tip of the catheter, pneumomediastinum and bilateral hydrothorax.

After surgical excision of the primary mass, the patient received high-dose chemotherapy in preparation for autologous stem cell reinfusion. During in-hospital infusion of busulfan, the patient presented neck swelling. A chest x-ray showed dislocated catheter and bilateral pleural effusion (Figure 2); the CT scan proved the tip of the catheter had transfixed the medial wall of the right jugular vein, entering the mediastinum. At the same time pneumomediastinum, subcutaneous neck emphysema and bilateral pleural effusion were noticed, and a thrombus was visible in the right jugular vein at the insertion in the brachiocephalic vein (Figure 3).

**Figure 4:**
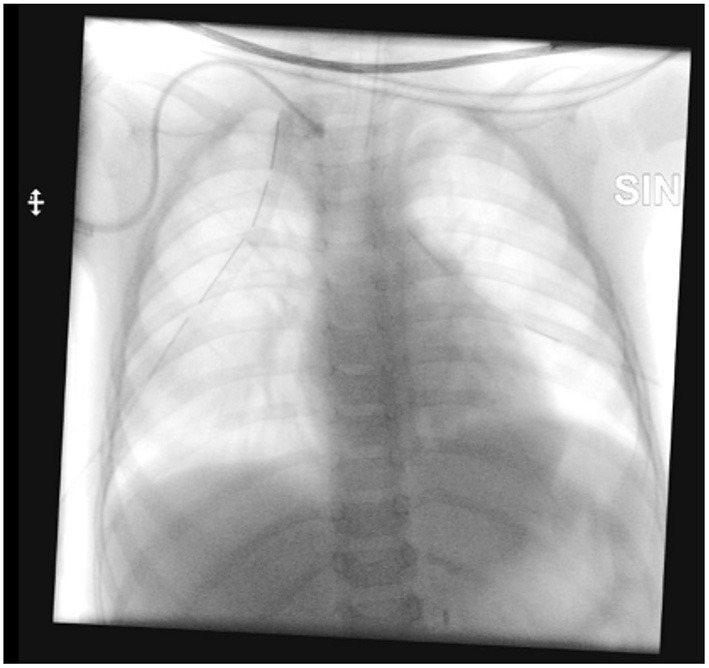
Removal of the dislocated central venous catheter.

Given the potential sudden deterioration of the clinical conditions, the patient was transferred to the intensive care unit and bilateral thoracostomy tubes were placed urgently. During the procedure, 500 mL of clear fluid were evacuated from pleural spaces (350 mL from the right, 150 mL from the left) with a Busulfan concentration of 786.88 ng/mL; after evacuation of busulfan solution, normal saline was flushed and aspirated repeatedly through thoracostomy tubes in order to remove any residue of cytotoxic drug. Once the patient had been stabilized, the dislocated catheter was removed electively on the following day under fluoroscopy (Figure 4).

Post-operative course was uneventful; the patient was transferred from Intensive Care Unit to the Oncology Department after 72 h. A new central venous catheter was placed, under ultra-sound guidance, in the left internal jugular vein 7 days after the removal of the previous catheter, without complications (Table 1).

**Table 1:** Timeframe of the relevant CVC-related events.

**Time**	**0**	**3 months**	**4 months**	**4 months + 1 day**	**4 months + 7 days**	**15 months**
Event	Diagnosis of high risk neuroblastoma	Last CT scan verifying correct position of CVC	Evidence of neck swelling	CVC removal under fluoroscopy	CVC replacement under ultra sound	Last follow up, no CVC related problems
	CVC placement under ultra sound		Rx and CT evidence of CVC dislodgement			
			Tube thoracostomy			

## Discussion

The use of long-term tunneled central venous catheters greatly facilitates the administration of cytotoxic and supportive therapy to children affected by malignancies (2, 8); on the other hand, these devices present the risk of insertion–related complications, which occur in 7–18% patients (9).

Malposition and unintentional pleural puncture are the two most common mechanical insertion-related problems; they mainly occur in the early post-procedural period and are most commonly associated with the use of the “landmark approach” (10), which consists in the use of anatomical landmarks on the body surface to guide the venipuncture, such as Sedillot's triangle on the base of the neck (8).

Late mechanical complications include occlusion, which is more likely with catheters placed in the subclavian vein and is usually related to thrombosis or pinch-off syndrome, and migration of the tip of the catheter, which may occur with trauma to catheter site or accidental pulling of the device (3).

Catheter placement techniques have improved in the recent decades, thanks to the use of ultra-sound to reduce the risk of malposition and pleural puncture (11–14).

In the present case, the patient had a central catheter inserted in the right internal jugular under ultra-sound guidance and fluoroscopic control, which is the most internationally recommended technique (2, 12); despite these measures, dislocation of the tip of the catheter occurred as a late event, probably due to right internal jugular vein thrombosis. Alternative explanations might be local trauma or accidental pulling of the line; however, no trauma nor pulling of the line were reported by parents nor by nursing staff. In addition, the securement device and the catheter cuff were not displaced at the time of removal.

The occurrence of bilateral pleural effusion in this patient is difficult to explain. Hydrothorax secondary to infusion extravasation has been reported as an early complication after the placement of the catheter; the underlying mechanism consists in the perforation of the lateral wall of superior vena cava and subsequent catheter tip misplacement in the thorax, both because of initial catheter malposition (15) or vascular erosion (16). In the present case, the bilateral hydrothorax occurred as a late complication and the vascular erosion occurred at the level of the internal jugular vein.

The cause of pneumomediastinum is uncertain. The tip of the catheter was far from trachea, as shown in the radiologic images (Figures 2, 3), which makes direct injury to airways extremely unlikely. Direct lesion to visceral pleura could be an explanation; in such case, hydropneumothorax would be present and suction should be applied through thoracostomy tubes to resolve pleural effusion. The absence of pneumothorax and the fact that thoracostomy tubes were effective even without active suction makes this explanation improbable. The most likely explanation is accidental infusion of air through the dislodged catheter.

We suppose that the thrombus in the distal part of the internal jugular vein may have progressively dislodged the tip of the vascular catheter, which eroded the medial wall of the vein in a proximal site; the solution infused through the dislodged catheter have then diffused into the mediastinal soft tissues and into the pleural cavities, even in the absence of a previous trauma of the pleura itself.

In conclusion, life-threatening central venous catheter-related mechanical complications may occur despite the use of all the state-of-the-art measure to reduce the risk of complications (i.e., polyurethane cuffed catheters, ultrasound-guided placement and subcutaneously anchored securement device) and after several months of uneventful use of the catheter itself; the present case highlights the importance of careful monitoring of patients with central venous catheters, especially when they develop unexpected symptoms or when catheters seem to be malfunctioning, in order to rapidly diagnose and promptly treat potentially lethal complications.

## Data availability statement

The original contributions presented in the study are included in the article/supplementary material, further inquiries can be directed to the corresponding author/s.

## Author contributions

TD'A: study design, data collection, and draft writing and editing. GPe: study design, literature search, and draft writing and editing. AC and AI: study supervision and draft revision. GPa and GN: technique description and draft revision. All authors contributed to the article and approved the submitted version.

## Conflict of interest

The authors declare that the research was conducted in the absence of any commercial or financial relationships that could be construed as a potential conflict of interest.

## Publisher's note

All claims expressed in this article are solely those of the authors and do not necessarily represent those of their affiliated organizations, or those of the publisher, the editors and the reviewers. Any product that may be evaluated in this article, or claim that may be made by its manufacturer, is not guaranteed or endorsed by the publisher.
